# Immersive Virtual Reality Medical Simulation: Autonomous Trauma Training Simulator

**DOI:** 10.7759/cureus.8062

**Published:** 2020-05-11

**Authors:** Kyle Couperus, Scott Young, Ryan Walsh, Christopher Kang, Carl Skinner, Robyn Essendrop, Kristin Fiala, Jillian F Phelps, Zachary Sletten, Matthew T Esposito, Jason Bothwell, Chad Gorbatkin

**Affiliations:** 1 Emergency Medicine, Madigan Army Medical Center, Tacoma, USA; 2 Emergency Medicine, Vanderbilt University Medical Center, Nashville, USA; 3 Emergency Medicine, University of Washington, Seattle, USA; 4 Military/Emergency Medicine, Uniformed Services University of the Health Sciences, Bethesda, USA; 5 College of Medicine, Baylor University, Houston, USA; 6 Emergency Medicine, Darnall Army Medical Center, Fort Hood, USA; 7 Emergency Medicine, San Antonio Military Medical Center, San Antonio, USA

**Keywords:** trauma, virtual reality training, virtual reality, role of virtual reality, simulator, medical education, educational technology, augmented reality, simulation, simulation-based medical education

## Abstract

Background

Medical and traumatic emergencies can be intimidating and stressful. This is especially true for early-career medical personnel.Training providers to respond effectively to medical emergencies before being confronted with a real scenario is limited by unnatural or high-cost training modalities that fail to realistically replicate the stress and gravity of real-world trauma management. Immersive virtual reality (IVR) may provide a unique training solution.

Methods

We created a working group of 10 active duty or former military emergency medicine physicians and two technical experts. We hosted 10 meetings to facilitate the development process. The program was developed with financial support from the Telemedicine and Advanced Technology Research Center (TATRC), through the primary vendor Exonicus, Inc, with support from Anatomy Next Inc, and Kitware, Inc. Development was completed using an agile project management style, which allowed our team to review progress and provide immediate feedback on previous milestones throughout its completion. The working group completed the resulting four simulation scenarios to evaluate perceived realism and training potential. Finally, testing of the technology platform off the network in a deployed role 3 was conducted.

Results

Upon completion, we created four IVR scenarios based on the highest mortality battlefield injuries: hemorrhage, tension pneumothorax, and airway obstruction. The working group unanimously indicated a high level of realism and potential training usefulness. Throughout this process, there have been a number of lessons learned and we present those here to show what we have created as well as provide guidance to others creating IVR training solutions.

Conclusion

Our team developed trauma scenarios that, to our knowledge, are the only IVR trauma scenarios to run autonomously without instructor input. Furthermore, we provide a potential template for the creation of future autonomous IVR training programs. This framework may offer a dynamic starting point as more teams seek to leverage the capabilities IVR offers.

## Introduction

Immersive virtual reality (IVR) can be highly effective as a medical simulation training platform [1-7]. Given recent advancements, this technology has become increasingly portable and visually realistic. While IVR technology appears to hold promise, there is a great deal to learn about the best way to functionally develop, implement, and share these training resources. Several commercial groups have created models that recreate current simulation lab environments with instructor input. While these systems increase training opportunities, decrease equipment needs, and offer broad potential, they still require a skilled trainer to ‘prompt the system’. Removing this limitation seems like a potential way to increase scalability. We are currently in the process of creating, to our knowledge, the only IVR simulator that would offer immediate autonomous feedback to users through both real-time patient physiologic responses and overall grading [8]. We will present phase 1 of this (multiphase) project. 

## Materials and methods

We created a working group of 10 active duty or former military emergency medicine physicians, and two technical experts who had programming and IVR project experience. We hosted 10 meetings to facilitate the development process (results). The project was completed at Madigan Army Medical Center in Tacoma, WA. The program was developed with financial support from the Telemedicine and Advanced Technology Research Center (TATRC), through the primary vendor Exonicus, Inc, with support from Anatomy Next Inc, and Kitware, Inc. Development was completed using an agile project management style, which allowed our team to review progress and provide immediate feedback on previous milestones throughout its completion. The working group completed the resulting four simulation scenarios to evaluate perceived realism and training potential. Finally, the technology platform was tested in a live, off the network, deployed environment in Iraq at a role 3 facility (deployed military hospital).

## Results

Upon completion of phase 1, we have created four IVR scenarios based on the highest mortality battlefield injuries: hemorrhage, tension pneumothorax, and airway obstruction. Throughout this process, there have been a number of lessons learned. We present those here to show what we have created as well as provide guidance to others creating IVR training solutions (summary shown in Table 1). 

**Table 1:** Development Process Summary HTC Vive (HTC, Taoyuan City, Taiwan), CAE (CAE Healthcare Inc, Montreal, Canada), Oculus Rift (Oculus VR, Irvine, CA), Magic Leap (Magic Leap, Inc., Plantation, FL)

Steps (Approximate Order)	Sample of Options
Select a training goal and simulation plan	Procedural trainer or decision trainer? Guided process or free-for-all/sandbox mode? Instructor-less systems may fit more algorithmic processes/procedures
Select a virtual reality platform	Microsoft Mixed Reality, Oculus Rift, HTC Vive, Magic Leap, phone-based system, many more
Select a physiology engine	BioGears, Pulse, CAE, HuMoD, many more
Develop the case	Based on prior decisions (above), available physiology engine, and specific capabilities of the selected immersive virtual reality platform, a case can be designed to meet your training goal
Create a master action list, grading scheme, and feedback plan	Based on the case and learning objective, create a list of every potential action a user can make in the environment. These can be tracked for feedback/grading
Create a required room and 3D objects list	Based on the case and required actions, the environment and three-dimensional objects list can be created
Visual, audio, and exam cues	Based on the case, review audio/visual/exam cues the learner will need to make a decision or complete a procedure. Be aware, there are some limitations representing physical exam findings in virtual reality (for example, palpation)
Team members	Determine what team members/additional characters will be required to complete the case/procedure. Nurse, assistant, medic, etc. Determine an interaction method, for example, point and click or verbal
User interface	Determine how the learner will interact with their environment/objects/patient/lab results/orders. For example, clicking, direct movement, verbal, or using an object (tablet/computer)
User tutorial	To work without an instructor, a guided walkthrough/tutorial is optimal
Gaze and location tracking	Optional tracking systems are available to track gaze, distance traveled, and a multitude of other variables
Development and feedback plan	Determine a method for providing the user with feedback on their performance. For example, ongoing during the case or in a summary at the end

Virtual reality platform

We reviewed the technical specifications of the current IVR platforms (Mobile, Microsoft Mixed Reality, HTC Vive), and augmented reality platforms (Hololens). A smaller focus group tried each platform and reviewed computing, graphics, network connectivity, and space requirements. Given its portability, graphics capabilities, and computing potential, we opted for primary development using Microsoft Mixed Reality.

Select a training goal/simulation plan

We initially sought to create a visually realistic training environment around one case. However, this seemed to limit immediate training benefit. Furthermore, the environment may be visually real, but most IVR platforms lack easy and scalable methods to change ambient temperature, moisture, or produce complex haptics. As we learned how to incorporate the physiology engine, we shifted towards a complex medical decision trainer. This could be placed in an endless number of environments and internally scaled to multiple patients in future iterations. The user is presented with an unstable trauma patient with a random injury. The physiology engine settings were selected to result in the patient’s death in 2-2.5 minutes if the player does not identify the injury and complete appropriate intervention. The player must keep the patient alive for a minimum of five minutes. Keeping the patient alive is the primary endpoint for the user. Additional factors, such as whether or not a complete assessment was performed, are tracked/graded, but do not affect the patient’s immediate survival. Standard trauma care actions are also available and tracked.

Selecting the case

Several individual cases, procedures, and environments were considered. However, selecting a single case or procedure seemed to drastically limit the scalability. We selected a generalizable trauma scenario for a few reasons. First, it allows multiple branch points to individual procedures (minisimulations) in future iterations. Second, several open-source physiology engines exist to run the physiology in these cases. The individual scenarios were further built to allow three progressive levels of consciousness if an injury is not addressed in time, and a failure state (death) that can be reversed if an injury is identified and treated.

The room and 3D objects

We sought to include every three-dimensional (3D) object that could be required in caring for a trauma patient, which totaled 36 items. The room was based on a 3D rendition of a standard military trauma bay.

Visual, audio, and exam cues

The selected injury scenario dictated visual/audio/exam cues (as in a traditional simulation).

Physiology engine

The incorporation of the physiology engine (Pulse) allowed us to develop a dynamic and more realistic simulation. Tying the simulation timeline to the physiology engine allows a player to see realistic vital sign changes, and complete the course of care as they would in a real patient scenario. It also allows for rapid case variations and randomization.

Team member interaction

Trauma management is a team sport. While a multiplayer/multidisciplinary approach is optimal, this again requires multiple skilled professionals to participate simultaneously. We sought to automate this process through two computer characters: a Nurse and a Medic. Commands are given vocally or through gaze-activated menus. 

Master action list/grading scheme/feedback system

A list of all the potential actions a player could make was developed (132), and each was tied to a specific outcome (injury treatment, lab availability, medication administration). These outcomes are injury and level of consciousness specific. Furthermore, each action was tied to a grade based on useful actions, neutral actions, and harmful actions. The Joint Trauma Committee Clinical Practice Guidelines and Advanced Trauma Life Support content were incorporated into the grading schematic.

User interface

The working group members have been end users of several computer training solutions, and sought to minimize technical frustration. The primary mode of interaction is using 3D objects to trigger animations. There are also multiple duplicate pathways, for example, starting intravenous access via a 3D object, voice command, or a menu selection.

User tutorial

It was readily apparent a thorough self-guided user tutorial would be necessary. We included key interventions and steps that would allow the user to ‘interact’ in the virtual environment.

Gaze/location tracking system

Given the user is completing actions in a digital world, it is quite easy to track multiple data points that may further relate to performance. We incorporated a gaze/location tracking system to allow for more analytics.

**Figure 1:**
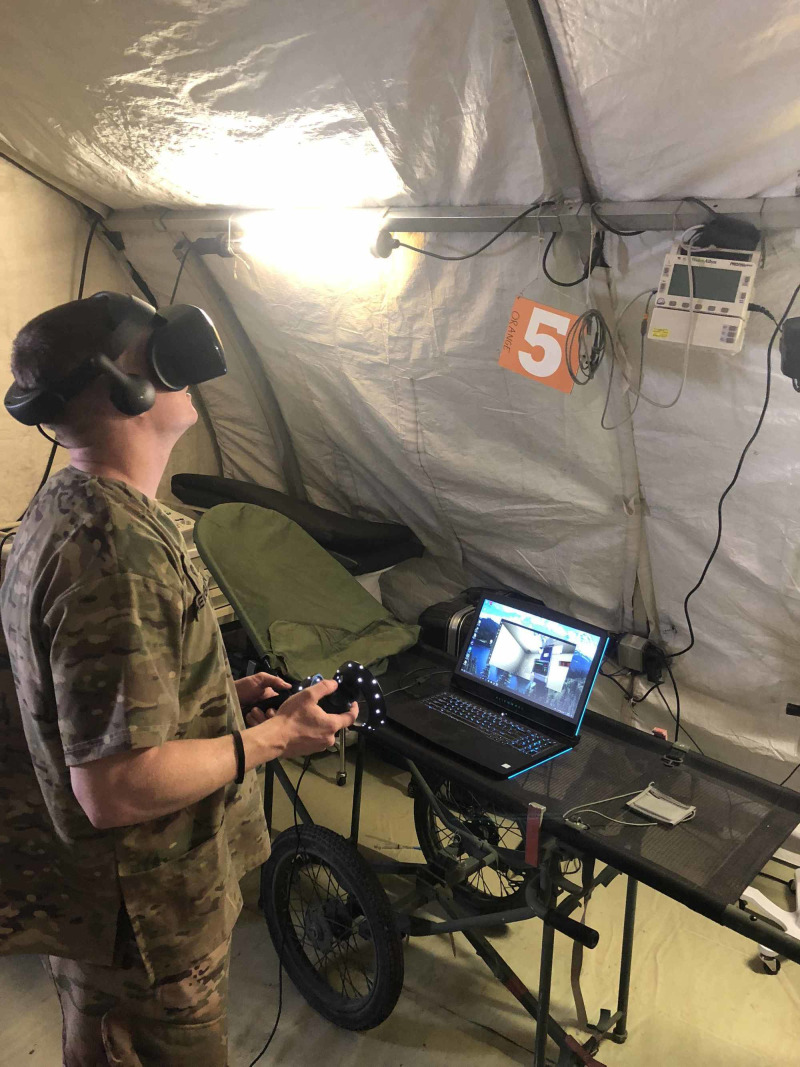
Medic completing an immersive virtual reality trauma simulation

Upon completion of the trauma scenario creation, the working group unanimously indicated a high level of realism and potential training usefulness. The technology platform worked in a deployed environment without internet connectivity (Figure 1), further highlighting the capabilities of this autonomous IVR system for military training. 

## Discussion

Our team developed four trauma scenarios that per our knowledge, after an extensive literature review, are the only IVR trauma scenarios to run autonomously without instructor input. Furthermore, we provide a potential template for the creation of future autonomous IVR training programs. This simulator, and IVR broadly, still has several limitations. First, the use of IVR can induce side effects (headaches, dizziness, and nausea). While the advancements in technology have greatly improved this, it is still an issue for some users. Second, it is challenging to create a seamless verbal feedback mechanism. Microsoft Mixed Reality does have voice recognition capabilities, but this produced mixed results when asking the simulated patient questions. Finally, the hardware and software are expensive. They both require initial development and upkeep. However, similar to computers, the cost of IVR systems and programming has been decreasing. Furthermore, these systems are still cheaper and more portable than most mannequin-based simulation systems. 

## Conclusions

Overall, this pilot project helps reveal the broad potential IVR has for medical training through automated and instructorless scenarios. These and similar self-directed scenarios may create a scalable simulation platform, similar to flight simulation training. We feel this holds immense learning and training potential for medical education. Several challenges still exist including side effects and high costs for development/equipment. Furthermore, more research is needed around learning retention, optimal content, and delivery curriculums. The framework presented in this article may offer a dynamic starting point as more teams seek to leverage IVR for medical education.
